# Long non-coding RNA HNF1A-AS1 promotes proliferation and suppresses apoptosis of bladder cancer cells through upregulating Bcl-2

**DOI:** 10.18632/oncotarget.20795

**Published:** 2017-09-08

**Authors:** Yonghao Zhan, Yifan Li, Bao Guan, Zicheng Wang, Ding Peng, Zhicong Chen, Anbang He, Shiming He, Yanqing Gong, Xuesong Li, Liqun Zhou

**Affiliations:** ^1^ Department of Urology, Peking University First Hospital, The Institute of Urology, Peking University, National Urological Cancer Centre, Beijing, 100034, China; ^2^ Department of Urology, State Engineering Laboratory of Medical Key Technologies Application of Synthetic Biology, Key Laboratory of Medical Reprogramming Technology, Shenzhen Second People's Hospital, The First Affiliated Hospital of Shenzhen University, Shenzhen, 518035, China

**Keywords:** bladder cancer, LncRNA, tumorigenesis, Bcl-2, HNF1A-AS1

## Abstract

Emerging evidences have indicated that long non-coding RNAs (lncRNAs) are pivotal regulators of tumor development and progression. HNF1A-AS1 (HNF1A antisense RNA 1, C12 or f27) is a novel long non-coding RNA that acts as a potential biomarker and is involved in development and progression of several cancers. Nevertheless, we know nothing about the clinical significance and molecular mechanism of HNF1A-AS1 in bladder cancer. In this study, we found that HNF1A-AS1 is significantly up-regulated in bladder cancer. Further experiments had demonstrated that silencing HNF1A-AS1 in bladder cancer cells could inhibit the proliferation and induce apoptosis. Mechanistically, we found down-regulated of HNF1A-AS1 increased the expression of miR-30b-5p and subsequently inhibited the expression of Bcl-2, in a ceRNA-dependent way. Moreover, knockdown of miR-30b-5p reversed cell proliferation inhibition and cell apoptosis induced by silencing HNF1A-AS1. In conclusions, we demonstrated that HNF1A-AS1 plays an important regulatory role in bladder cancer and shed new light on lncRNA-directed diagnostic and therapeutics in bladder cancer.

## INTRODUCTION

Bladder cancer is the ninth most common malignancy all over the world and it is estimated that there are 386,000 new cases are diagnosed worldwide annually [1–4]. Despite improvements in current clinical treatment such as surgery, radiation therapy, and chemotherapy, the overall survival (OS) time of bladder cancer patients has not been significantly improved [5–8]. The prognosis of bladder cancer is closely related to the stage of disease, but patients do not have specific symptoms at the early stage of bladder cancer [9–12]. Therefore, novel markers for diagnosis at early stage and more efficient and safer therapeutic method are urgently needed [13, 14].

The long non-coding RNAs (lncRNAs) are important new members of the ncRNA family with greater than 200 nucleotides in length [15, 16]. The rapid development of RNA genomics has highlighted the role of long non-coding RNAs (lncRNAs) in many human diseases, especially in cancers [17, 18]. Recent evidences have showed that many lncRNAs play important regulatory roles in diverse biological processes of cancers, such as UCA-1, HOTAIR, H19, MALAT1, ZEB1-AS1, PVT-1, PANDAR and etc [19–25]. HNF1A-AS1 (HNF1A antisense RNA 1) is a novel identified lncRNA with 2455 nucleotides in length and localized at the chromosome 12 [26]. Recently, HNF1A-AS1 originally was identified as a potential biomarker and was involved in the development of multiple cancers [26–29]. However, the biological function and underlying mechanism of action of HNF1A-AS1 in bladder cancer is completely unknown.

In the present study, we found that HNF1A-AS1 is significantly up-regulated in bladder cancer. We demonstrated that silencing HNF1A-AS1 could significantly inhibit proliferation and induce apoptosis in bladder cancer cells. Emerging evidences have indicated that lncRNAs regulate gene expression at different processing levels, including chromatin modification, transcription and posttranscriptional regulation [30–32]. In our study, we found HNF1A-AS1 could directly interact with miR-30b-5p and down-regulation of HNF1A-AS1 increased the expression of miR-30b-5p and subsequently inhibited the expression of Bcl-2. Thus, HNF1A-AS1 positively regulated the expression of Bcl-2 through sponging miR-30b-5p, and played an important regulatory role in bladder cancer progression. Cumulatively, these findings demonstrate that HNF1A-AS1 is a key regulator in bladder cancer progression, which highlights its potential clinical utility as a promising prognostic and therapeutic target of bladder cancer.

## RESULTS

### HNF1A-AS1 was up-regulated in bladder cancer

**Figure 1:**
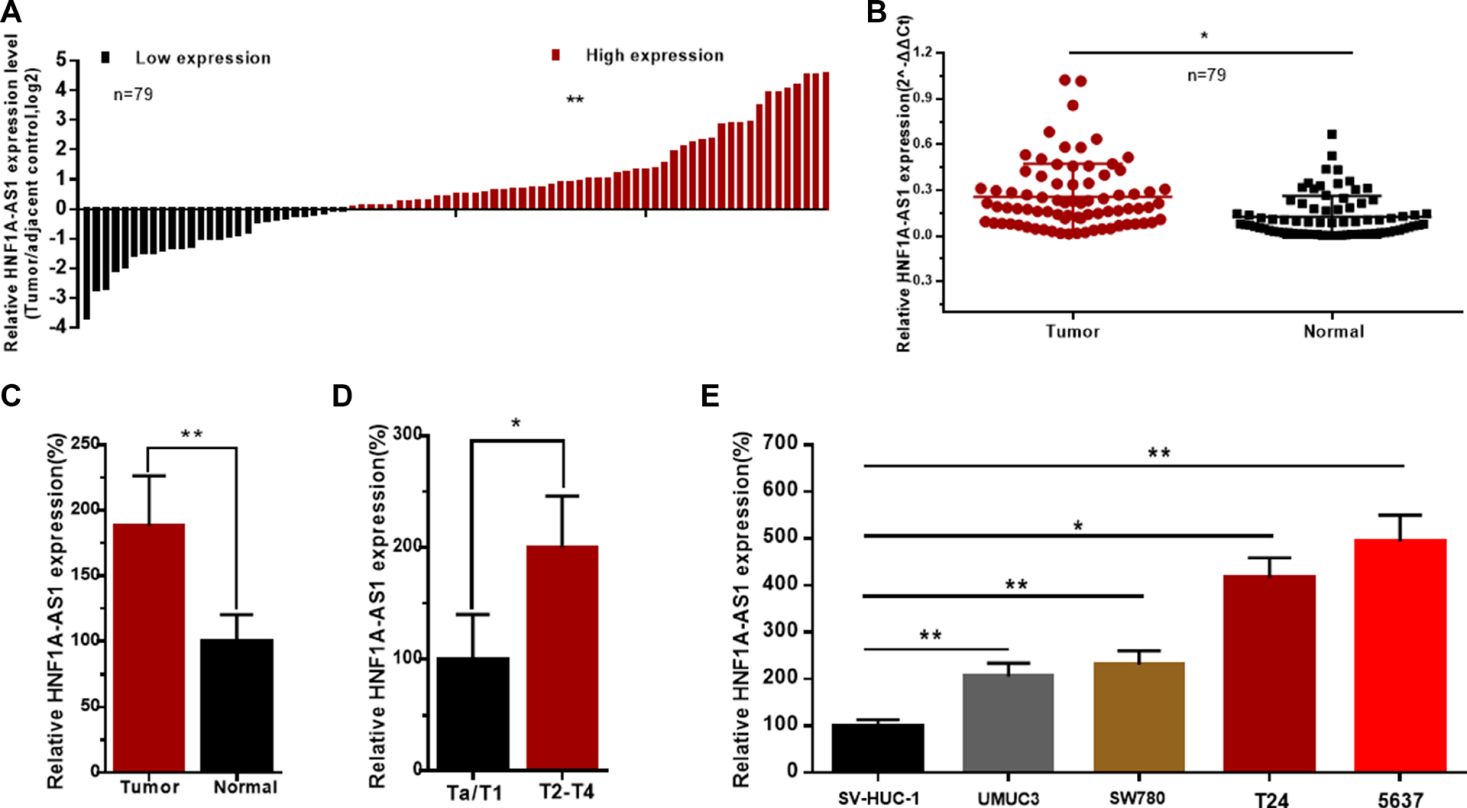
The relative expression levels of HNF1A-AS1 in bladder cancer The relative expression levels of HNF1A-AS1 were detected using qRT-PCR. (**A**) The heights of the columns in the chart represent the log2-transformed fold changes (bladder cancer tissue/normal bladder tissue) in HNF1A-AS1 expression in 79 patients with bladder cancer. (**B** and **C**) HNF1A-AS1 expression levels were higher in bladder cancer tissues than that in corresponding normal tissues. (**D**) HNF1A-AS1 expression was significantly higher in patients with advanced TNM stage. (**E**) HNF1A-AS1 was up-regulated in bladder cancer cell lines compared to normal urothelial cell line. Data are shown as mean ± SD.

The relative expression level of HNF1A-AS1 was determined by qRT-PCR in a total of 79 patients with urothelial bladder cancer. The HNF1A-AS1 expression fold change (bladder cancer tissue/matched normal tissue) in each patient was indicated in Figure 1A. As shown in Figure 1B, 1C, HNF1A-AS1 was up-regulated in bladder cancer tissues compared to corresponding non-tumor tissues. Moreover, up-regulated HNF1A-AS1 expression was positively correlated with advanced TNM stage (Figure 1D). HNF1A-AS1 was up-regulated in bladder cancer cell lines compared to corresponding normal urothelial cell line SV-HUC-1 (Figure 1E). These results demonstrated that HNF1A-AS1 may play an key role in bladder cancer. Clinicopathological features of patients and statistical results are shown in Table 1 and Supplementary Table 1, respectively.

**Table 1:** Correlation between HNF1A-AS1 expression and clinicopathological features of UCB patients

Parameters Total	Group	Total	HNF1A-AS1 expression		*P* value
**High**	**Low**
Gender	Male	57 (72%)	37 (47%)	20 (25%)	0.915
	Female	22 (28%)	14 (18%)	8 (10%)	
Age (years)	< 60	28 (35%)	17 (22%)	11 (13%)	0.597
	≥ 60	51 (65%)	34 (43%)	17 (22%)	
Tumor size (cm)	< 3 cm	32 (41%)	17 (22%)	15 (19%)	0.081
	≥ 3 cm	47 (59%)	34 (43%)	13 (16%)	
Multiplicity	Single	43 (55%)	24 (31%)	19 (24%)	0.076
	Multiple	36 (45%)	27 (34%)	9 (11%)	
Histological grade	L	35 (44%)	17 (21%)	18 (23%)	0.008**
	H	44 (56%)	34 (43%)	10 (13%)	
Tumor stage T	Ta,T1	22 (28%)	9 (11%)	13 (17%)	0.034*
	T2-T4	57 (72%)	42 (53%)	15 (19%)	
Lymph nodes metastasis	NO	74 (94%)	47 (59%)	27 (35%)	0.651
	YES	5 (6%)	4 (5%)	1 (1%)	

*P < 0.05 was considered significant (Chi-square test between 2 groups).

### Corresponding specific siRNA/pcDNA3.1 down/up-regulated expression level of HNF1A-AS1

**Figure 2:**
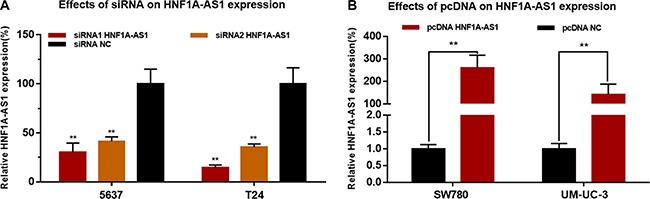
Effects of corresponding siRNA or pcDNA on HNF1A-AS1 expression level The relative expression level was determined using qRT-PCR. (**A**) The HNF1A-AS1 specific siRNA significantly down-regulated the expression level of HNF1A-AS1 in 5637 and T24 cells. (**B**) The HNF1A-AS1 specific pcDNA3.1 significantly up-regulated the expression level of HNF1A-AS1 in SW780 and UM-UC-3 cells. Data are indicated as mean ± SD.

Bladder cancer cells were cultured and transfected with HNF1A-AS1 specific siRNA (siRNA1/2 HNF1A-AS1) or HNF1A-AS1 expression vector (pcDNA3.1 HNF1A-AS1). The results showed that the relative expression level of HNF1A-AS1 in bladder cancer cells was significantly down-regulated by the siRNA HNF1A-AS1 (Figure 2A) and up-regulated by the pcDNA3.1 HNF1A-AS1 (Figure 2B).

### Silencing HNF1A-AS1 inhibited cell proliferation and overexpressing HNF1A-AS1 promoted cell proliferation

**Figure 3:**
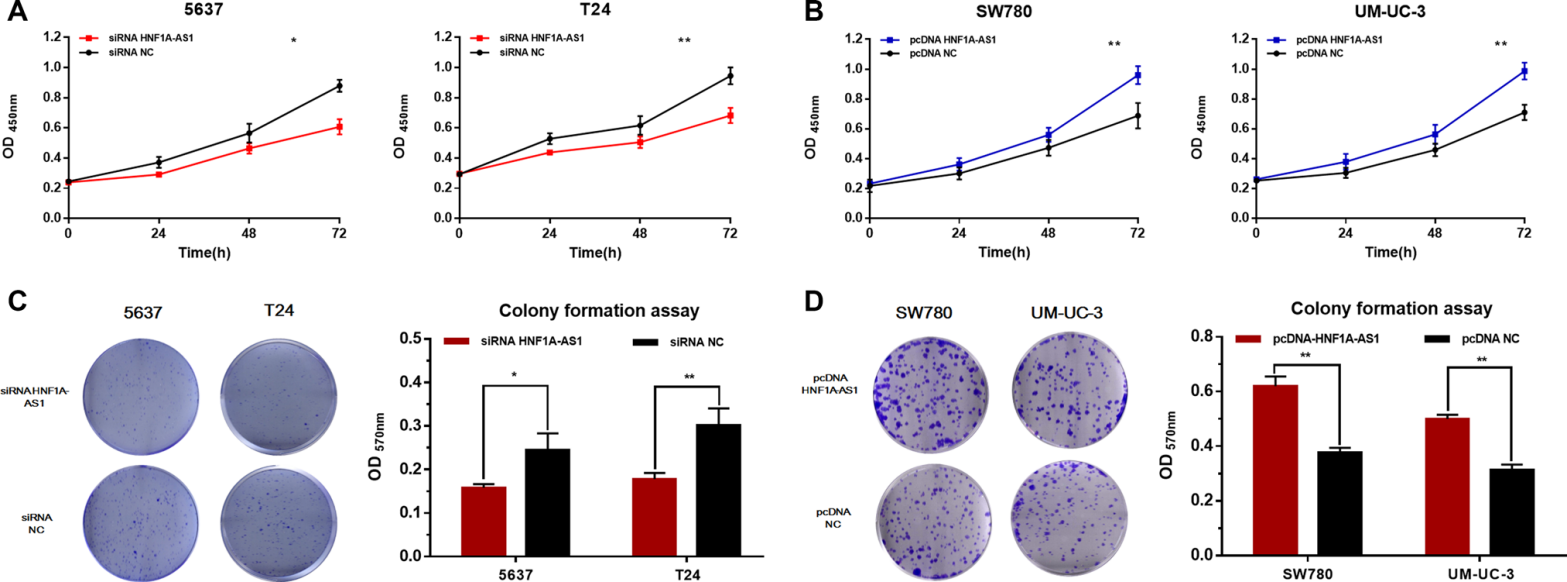
Effects of down-regulation or up-regulation of HNF1A-AS1 on cell proliferation Cell proliferation was determined by CCK-8 assay and colony formation assay. (**A** and **C**) Cell proliferation inhibition was observed in bladder cancer 5637 and T24 cells. (**B** and **D**) Cell proliferation promotion was observed in bladder cancer SW780 and UM-UC-3 cells. Data are shown as mean ± SD.

**Figure 4:**
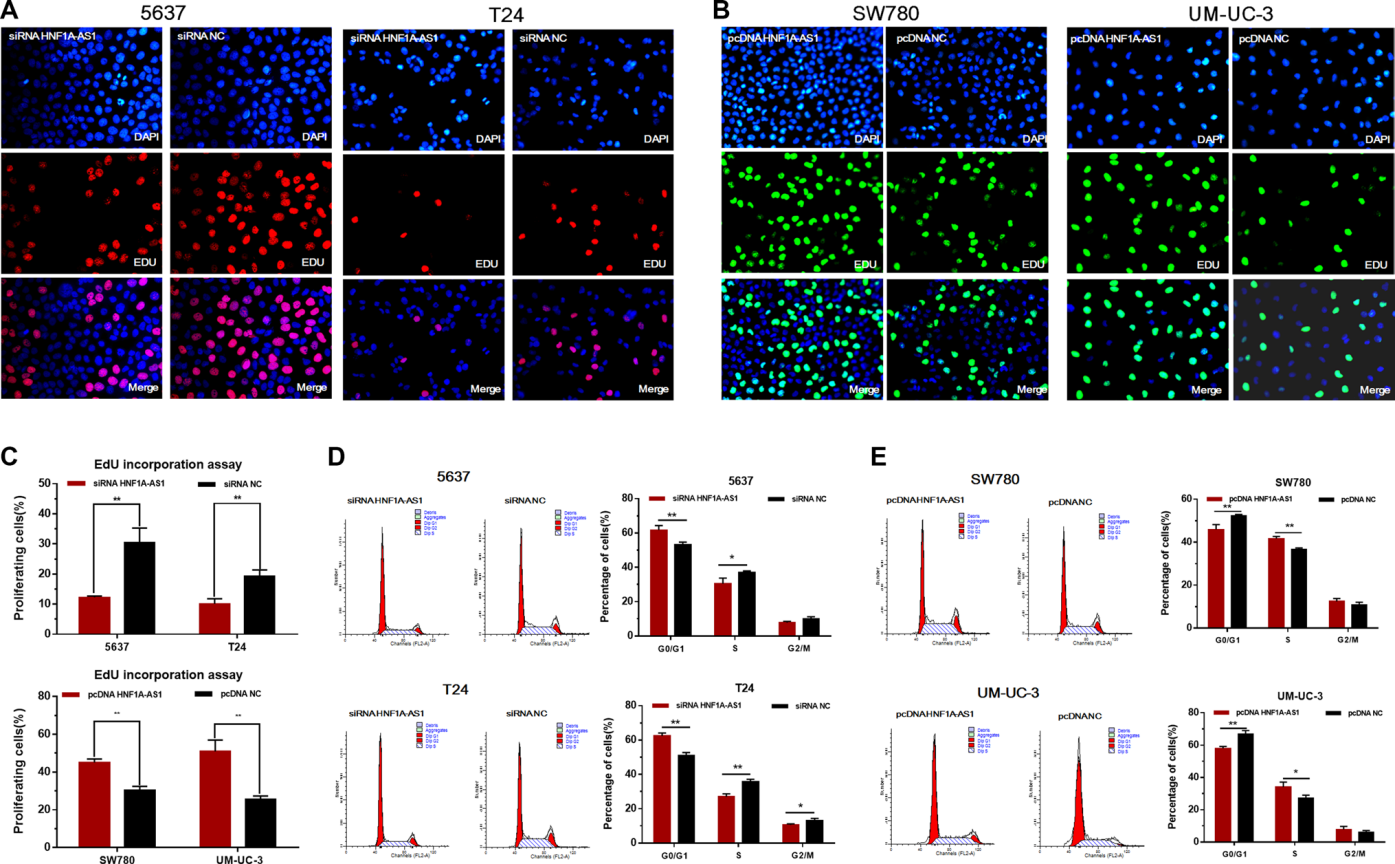
Effects of down-regulation or up-regulation of HNF1A-AS1 on cell proliferation Cell proliferation was also determined by Edu assay and flow cytometry. (**A**, **C** and **D**) Cell proliferation inhibition was observed in bladder cancer 5637 and T24 cells. (**B**, **C** and **E**) Cell proliferation promotion was observed in bladder cancer SW780 and UM-UC-3 cells. Data are shown as mean ± SD.

The cell proliferation changes of bladder cells were determined using CCK-8 assay, Colony formation assay, Flow cytometry and Edu assay. Inhibited cell proliferation was observed in 5637 and T24 cells (Figures 3A, 3C and Figure 4A, 4C, 4D) by silencing HNF1A-AS1. The promotion of cell proliferation was observed in SW780 and UM-UC-3 cells (Figure 3B, 3D and Figure 4B, 4C, 4E) by overexpressing HNF1A-AS1. These results demonstrated that HNF1A-AS1 promotes cell proliferation in bladder cancer.

### Silencing HNF1A-AS1 induced cell apoptosis and overexpressing HNF1A-AS1 suppressed cell apoptosis

**Figure 5:**
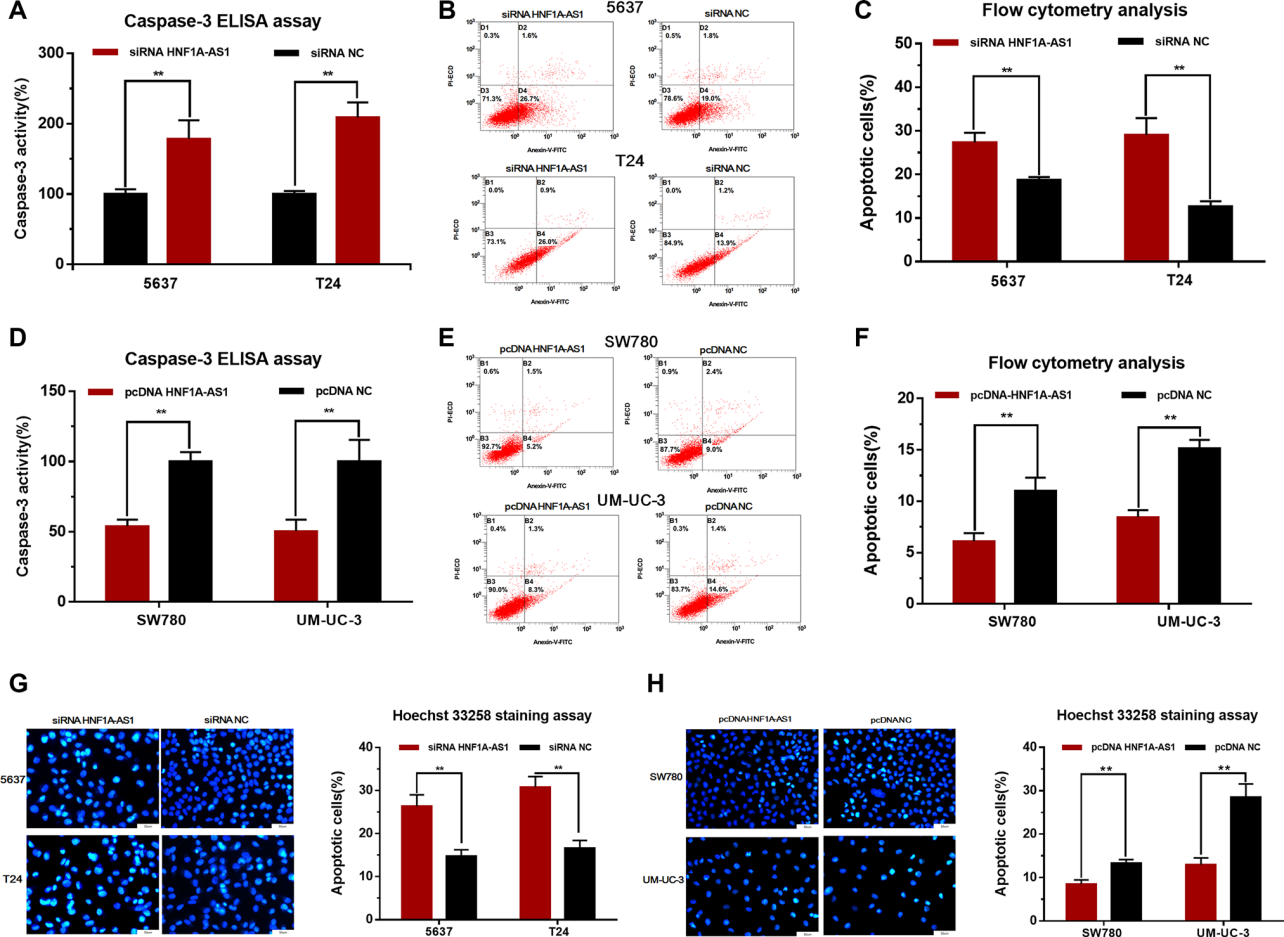
Effects of down-regulation or up-regulation of HNF1A-AS1 on cell apoptosis Cell apoptosis was determined by ELISA assay, Hoechst 33342 staining assay and Flow cytometry. (**A**, **B**, **C** and **G**) Induced cell apoptosis by silencing HNF1A-AS1 was observed in bladder cancer 5637 and T24 cells. (**D**, **E**, **F**, and **H**) Suppressed cell apoptosis by overexpressing HNF1A-AS1 was observed in bladder cancer SW780 and UM-UC-3 cells. Data are shown as mean ± SD.

The cell apoptosis changes of bladder cells were determined using ELISA assay, Hoechst 33342 staining assay and Flow cytometry. Induced cell apoptosis was observed in 5637 and T24 cells by silencing HNF1A-AS1 (Figure 5A–5CA, 5G). Suppressed cell apoptosis was observed in SW780 and UM-UC-3 cells (Figure 5D–5FD, 5H) by overexpressing HNF1A-AS1. These results demonstrated that HNF1A-AS1 suppresses cell apoptosis in bladder cancer.

### Silencing HNF1A-AS1 increased the expression of miR-101-3p and inhibited the expression of Bcl-2

**Figure 6:**
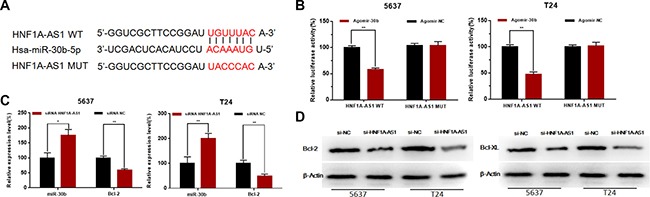
Effects of HNF1A-AS1 on the expression levels of miR-30b-5p and Bcl-2 (**A**) Bio-information analysis predicted that HNF1A-AS1 had putative binding sites with miR-30b. (**B**) HNF1A-AS1-WT and Agomir30b co-transfection significantly inhibited luciferase activity, and HNF1A-AS1-MUT and Agomir30b co-transfection failed to change luciferase activity in 5637 and T24 cell lines. (**C**) Down-regulated of HNF1A-AS1 increased the expression of miR-30b-5p and inhibited the expression of Bcl-2 mRNA. (**D**) Down-regulated of HNF1A-AS1 inhibited the protein expression of Bcl-2. Data are shown as mean ± SD.

To investigate the underlying mechanisms of HNF1A-AS1-mediated biological processes, we performed bio-information analysis, dual-luciferase reporter assay, qRT-PCR and western blotting analysis. First, through searching in online bioinformatics database, bio-information analysis predicted that HNF1A-AS1 had putative binding sites with miR-30b (Figure 6A). Detailed prediction results were shown in Supplementary Table 3. Dual-luciferase reporter assay showed HNF1A-AS1-WT and Agomir30b co-transfection significantly inhibited luciferase activity, and HNF1A-AS1-MUT and Agomir30b co-transfection failed to change luciferase activity in 5637 and T24 cell lines (Figure 6B). Western blotting analysis and qRT-PCR showed down-regulated of HNF1A-AS1 increased the expression of miR-30b-5p and subsequently inhibited the expression of Bcl-2 (Figure 6C and 6D).

### Knockdown of miR-30b-5p reversed cell proliferation inhibition and apoptosis induced by silencing HNF1A-AS1

**Figure 7:**
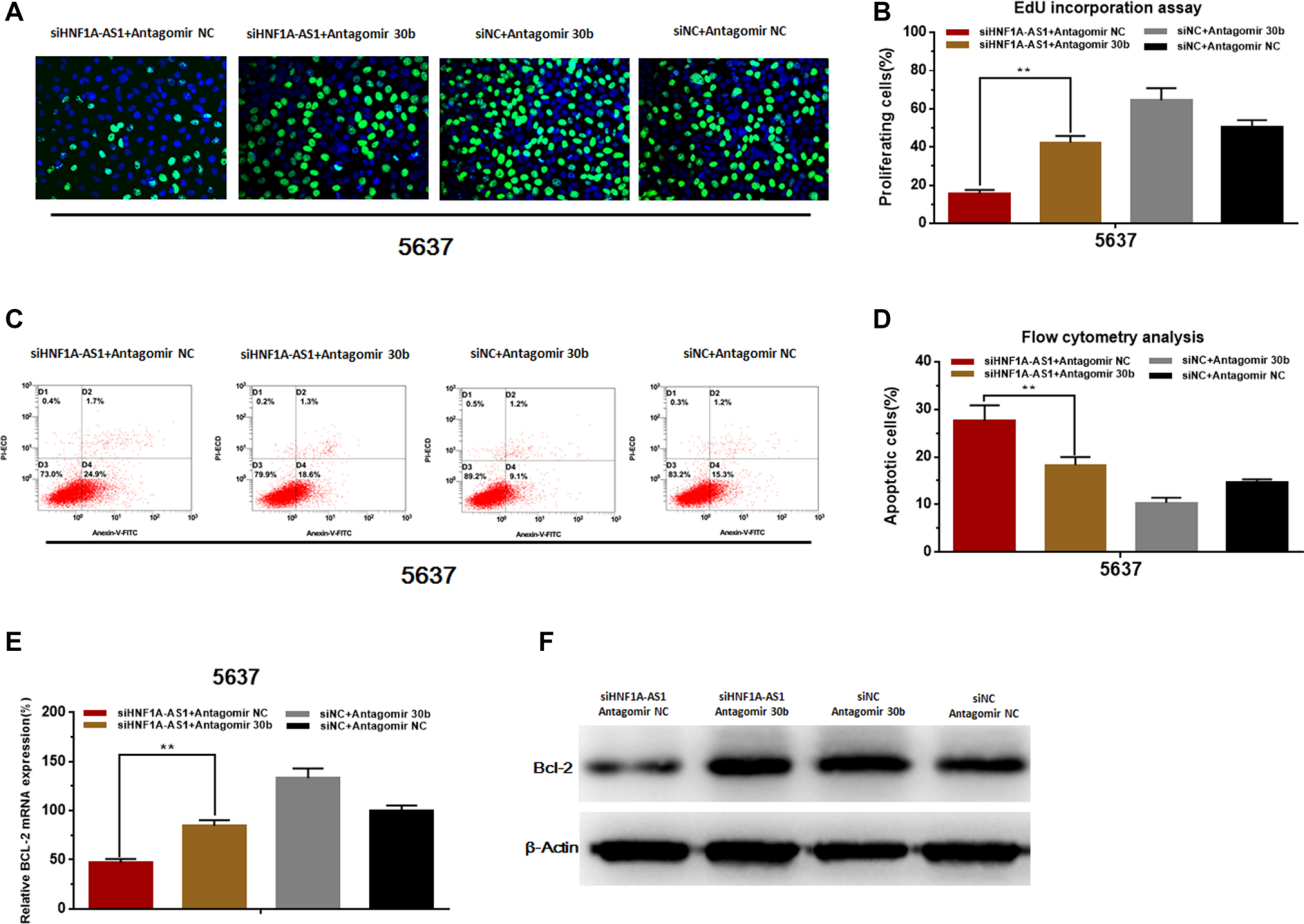
Knockdown of miR-30b-5p reverses cell proliferation inhibition and apoptosis induced by silencing HNF1A-AS1 (**A** and **B**) Knockdown of miR-30b-5p partly reversed cell proliferation inhibition induced by silencing HNF1A-AS1. (**C** and **D**) Knockdown of miR-30b-5p partly reversed cell apoptosis induced by silencing HNF1A-AS1. (**E** and **F**) Knockdown of miR-30b-5p partly reversed the suppression of Bcl-2 induced by silencing HNF1A-AS1. Data are shown as mean ± SD.

The cell proliferation changes of bladder cells were determined using Edu assay. The cell apoptosis changes of bladder cells were determined using flow cytometry. Knockdown of miR-30b-5p with antagomir-30b-5p partly reversed cell proliferation inhibition induced by silencing HNF1A-AS1 (Figure 7A, 7B). Knockdown of miR-30b-5p with antagomir-30b-5p partly reversed cell apoptosis induced by silencing HNF1A-AS1 (Figure 7C, 7D). Through analyzing the western blotting and qRT-PCR results, we found that knowdown of miR-30b-5p could partly reverse the suppression of Bcl-2 which was induced by silencing HNF1A-AS1 (Figure 7E, 7F).

## DISCUSSION

Bladder cancer is the most common urologic tumors in China and its incidence and mortality have been significantly increased in the past decade [33, 34]. There are no specific symptoms for patents with bladder cancer at the early stage, therefore, some bladder cancers are found at advanced stage when treatments are less effective [35]. Therefore, finding new prognostic and therapeutic target has enormous potential significance to improving the clinical strategies and outcomes of bladder cancer [36–38].

The lncRNAs are important new members of no-coding RNA family, which are longer than 200 nucleotides [39–41]. Recently, an increasing number of evidences indicated that lncRNAs play important roles in cancer occurrence and progression [42–44]. The lncRNA HNF1A-AS1 has been reported to interact with HNF1A and H19 to regulate oesophageal adenocarcinoma occurrence and progression [26]. And HNF1A-AS1 has also been reported to mediate the binding of DNMT1 to E-cadherin in lung adenocarcinoma [28]. We found that HNF1A-AS1 was significantly up-regulated in bladder cancer and silencing HNF1A-AS1 significantly inhibited proliferation and induced apoptosis in bladder cancer cells. Mechanistically, although HNF1A-AS1 had been reported to mediate the binding of DNMT1 to E-cadherin in lung adenocarcinoma, we did not found HNF1A-AS1 interact with DNMT1 and E-cadherin in bladder cancer [28]. Different from other cancers, we found HNF1A-AS1 could directly interact with miR-30b-5p. The down-regulation of HNF1A-AS1 could increase the expression of miR-30b-5p and subsequently inhibit the expression of Bcl-2, in a ceRNA-dependent way. Moreover, we performed reverse experiments and found knockdown of miR-30b-5p reversed cell proliferation inhibition, cell apoptosis and the suppression of Bcl-2 induced by silencing HNF1A-AS1.

Cumulatively, these findings demonstrated that HNF1A-AS1 is a key regulator in bladder cancer progression and may serve as a potential diagnostic and therapeutic target of bladder cancer.

## MATERIALS AND METHODS

### Patients and clinical samples collection

A total of 79 patients with urothelial carcinoma of bladder who received radical or partial cystectomy were included in this study. After resection fresh bladder cancer tissue and pair-matched normal adjacent bladder tissue obtained from the same patient were snap-frozen in liquid nitrogen immediately. All patients included in this study signed informed consent and this study was approved by the Institutional Review Board of Peking University First Hospital, Beijing, China and Shenzhen Second People's Hospital, Shenzhen, China.

### Bladder cancer cell lines and cell culture

Bladder cancer 5637, SW780, UMUC3, T24 and SV-HUC-1 cells used in this study were purchased from the Institute of Cell Research, Chinese Academy of Sciences, Shanghai, China. The UMUC3, T24 and SV-HUC-1 cells were cultured in Dulbecco's Modified Eagle Medium (Invitrogen, Carlsbad, CA, USA) plus 10% fetal bovine serum. The 5637 cells and SW780 cells were cultured in RPMI-1640 Medium (Invitrogen, Carlsbad, CA, USA) plus 10% fetal bovine serum. Corresponding plates were placed at 37°C with a humidified atmosphere of 5% CO_2_ in incubator.

### siRNA and pcDNA transfection

The siRNA (small interfering RNA) sequences were as follows: siRNA1-HNF1A-AS1 (5′-CACCUG CAUUCAAACUCGGACUGUU-3′), siRNA2-HNF1A-AS1 (5′-GGGUGAGCAGCUGUUUGCAAGACUA-3′). Before transfection, the cells were cultured 24 h. Then, the cells were transiently transfected with corresponding siRNA or pcDNA using Lipofectamine 3000 Transfection Reagent (Invitrogen, Carlsbad, CA, USA) according to the manufacturer's instructions. After 48 h, cells transfected with corresponding siRNA or pcDNA were harvested for quantitative real-time PCR.

### RNA extraction and quantitative real-time PCR

The total RNA of the tissue samples and the transfected cells were extracted using the Trizol reagent (Invitrogen, Carlsbad, CA, USA) according to the manufacturer's instructions. The detailed primer sequences included in this study are shown in Supplementary Table 2. Quantitative real-time PCR was performed using the ABI PRISM 7000 Fluorescent Quantitative PCR System (Applied Biosystems, Foster City, CA, USA) according to the manufacturer's instructions and normalized to *β*-actin or U6 small nuclear RNA. Experiments were repeated at least three times.

### Cell counting Kit-8 assay

Cell proliferation was determined using Cell Counting Kit-8 (Beyotime Inst Biotech, China) according to the manufacturer's instructions. Briefly, 5 × 10^3^ cells/well were seeded in a 96-well flat-bottomed plate, and grown at 37°C for 24 h, then transfected with corresponding siRNA or pcDNA. Finally, the absorbance was finally determined at a wavelength of 450 nm using a microplate reader (Bio-Rad, Hercules, CA, USA). Experiments were repeated at least three times.

### Colony formation assay

Cell proliferation was determined using colony formation assay. 24 h after transfection, 2 × 10^3^ cells were seeded in a 6-well plate. The cells were incubated for 7d at 37°C, and then the cells were stained with 0.5%crystal violet solutionin 20% methanol. Then the absorbance were determined at a wavelength of 570 nm using an microplate reader (Bio-Rad, Hercules, CA, USA). Experiments were repeated at least three times.

### Ethynyl-2-deoxyuridine (EdU) incorporation assay

Cell proliferation was also determined by Ethynyl-2-deoxyuridine incorporation assay using an EdU Apollo DNA *in vitro* kit (RIBOBIO, Guangzhou, China) following the manufacturer's instructions. Briefly, after transfected with corresponding siRNA or pcDNA cells were incubated with 100 *μl* of 50 *μ*M EdU per well for 2 h at 37°C, respectively. Finally, the cells were visualized under a *fluorescence microscopy.* Experiments were repeated at least three times.

### Cleaved Caspase-3 ELISA assay

Cell apoptosis was determined by ELISA assay. Briefly, 5 × 10^5^ cells/well were seeded in a 6-well plate, and grown at 37°C for 24 h, then transfected with corresponding siRNA or pcDNA, respectively. At 48 h after transfection, Cell cleaved caspase-3 activity was measured using the Caspase-3 Colorimetric Assay kit (Abcam, Cambridge, UK) according to the manufacturer's instructions. Experiments were repeated at least three times.

### Hoechst 33342 staining assay

Cell apoptosis was also determined by Hoechst 33258 staining assay. At 48 h after transfection with corresponding siRNA or pcDNA, apoptotic cells were also observed by using the Hoechst 33258 staining kit (Life, Eugene, OR, USA) according to the manufacturer's instructions. Experiments were repeated at least three times.

### Flow cytometry analysis assay

Cell apoptosis and cell cycle were determined by Flow cytometry. Briefly, cells were cultured in normal medium and transfected with corresponding siRNA or pcDNA, respectively. Cells were collected after transfection for 48 h. Cell apoptosis was determined by PE Annexin V apoptosis detection kits (BD Pharmingen, San Diego, CA, USA). Cell cycle analysis was performed adopting propidium iodide cell cycle detected kits (BD Pharmingen). Finally, cell apoptosis and cell cycle were determined using flow cytometry (EPICS, XL-4, Beckman, CA, USA). Experiments were repeated at least three times.

### Dual-luciferase reporter assay

HNF1A-AS1-WT/MUT (GenePharma, Shanghai, China) were constructed and transfected into 5637 and T24 cell lines along with Agomir30b/NC. Luciferase activity was detected using the Dual-Luciferase Reporter Assay System (Promega; 48 h after transfection) according to the manufacturer's instructions. Experiments were repeated at least three times.

### Western blotting analysis

Total cell lysates were prepared in a 1× sodium dodecyl sulfate buffer. Total protein was separated by sodium dodecyl sulfate-polyacrylamide gel electrophoresis and transferred onto nitrocellulose membranes. Then the membrane was blocked with 5% non-fat milk and incubated with primary antibodies at 4°C overnight. After incubation with antibodies specific for Bcl-2/Bcl-XL/β-actin (Abcam, Hong Kong, China), the blots were incubated with goat anti-rabbit secondary antibody (Abcam, Hong Kong, China) and visualized with enhanced chemiluminescence. Experiments were repeated at least three times.

### Statistical analyses

All experimental data from three independent experiments were analyzed by Student's *t*-test or *χ^2^* test and results were expressed as mean ± standard deviation. *P*-values of less than 0.05 were considered to be statistically significant. All statistical tests were conducted by SPSS version 19.0 software (SPSS Inc. Chicago, IL, USA).

## SUPPLEMENTARY MATERIALS TABLES



## References

[1] Burger M, Catto JW, Dalbagni G, Grossman HB, Herr H, Karakiewicz P, Kassouf W, Kiemeney LA, La Vecchia C, Shariat S, Lotan Y (2013). Epidemiology and risk factors of urothelial bladder cancer. Eur Urol.

[2] Cancer Genome Atlas Research Network (2014). Comprehensive molecular characterization of urothelial bladder carcinoma. Nature.

[3] Chamie K, Ballon-Landa E, Bassett JC, Daskivich TJ, Leventhal M, Deapen D, Litwin MS (2015). Quality of diagnostic staging in patients with bladder cancer: a process-outcomes link. Cancer.

[4] Chamie K, Litwin MS, Bassett JC, Daskivich TJ, Lai J, Hanley JM, Konety BR, Saigal CS (2013). Urologic Diseases in America Project. Recurrence of high-risk bladder cancer: a population-based analysis. Cancer.

[5] Chou R, Selph SS, Buckley DI, Gustafson KS, Griffin JC, Grusing SE, Gore JL (2016). Treatment of muscle-invasive bladder cancer: A systematic review. Cancer.

[6] Galsky MD, Hendricks R, Svatek R, Bangs R, Hoffman-Censits J, Clement J, Dreicer R, Guancial E, Hahn N, Lerner SP, O'Donnell PH, Quale DZ, Siefker-Radtke A (2013). Critical analysis of contemporary clinical research in muscle-invasive and metastatic urothelial cancer: a report from the Bladder Cancer Advocacy Network Clinical Trials Working Group. Cancer.

[7] Powles T, Eder JP, Fine GD, Braiteh FS, Loriot Y, Cruz C, Bellmunt J, Burris HA, Petrylak DP, Teng SL, Shen X, Boyd Z, Hegde PS (2014). MPDL3280A (anti-PD-L1) treatment leads to clinical activity in metastatic bladder cancer. Nature.

[8] Zhan Y, Liu Y, Lin J, Fu X, Zhuang C, Liu L, Xu W, Li J, Chen M, Zhao G, Huang W, Cai Z (2015). Synthetic Tet-inducible artificial microRNAs targeting beta-catenin or HIF-1alpha inhibit malignant phenotypes of bladder cancer cells T24 and 5637. Sci Rep.

[9] Choi W, Porten S, Kim S, Willis D, Plimack ER, Hoffman-Censits J, Roth B, Cheng T, Tran M, Lee IL, Melquist J, Bondaruk J, Majewski T (2014). Identification of distinct basal and luminal subtypes of muscle-invasive bladder cancer with different sensitivities to frontline chemotherapy. Cancer Cell.

[10] Christodouleas JP, Baumann BC, He J, Hwang WT, Tucker KN, Bekelman JE, Tangen CM, Lerner SP, Guzzo TJ, Malkowicz SB, Herr H (2014). Optimizing bladder cancer locoregional failure risk stratification after radical cystectomy using SWOG 8710. Cancer.

[11] Casadio V, Molinari C, Calistri D, Tebaldi M, Gunelli R, Serra L, Falcini F, Zingaretti C, Silvestrini R, Amadori D, Zoli W (2013). DNA Methylation profiles as predictors of recurrence in non muscle invasive bladder cancer: an MS-MLPA approach. J Exp Clin Cancer Res.

[12] Feng Y, Liu J, Kang Y, He Y, Liang B, Yang P, Yu Z (2014). miR-19a acts as an oncogenic microRNA and is up-regulated in bladder cancer. J Exp Clin Cancer Res.

[13] Lerner SP, Goh A (2015). Novel endoscopic diagnosis for bladder cancer. Cancer.

[14] Vickers AJ, Bennette C, Kibel AS, Black A, Izmirlian G, Stephenson AJ, Bochner B (2013). Who should be included in a clinical trial of screening for bladder cancer? a decision analysis of data from the Prostate. Lung, Colorectal and Ovarian Cancer Screening Trial. Cancer.

[15] Kallen AN, Zhou XB, Xu J, Qiao C, Ma J, Yan L, Lu L, Liu C, Yi JS, Zhang H, Min W, Bennett AM, Gregory RI (2013). The imprinted H19 lncRNA antagonizes let-7 microRNAs. Mol Cell.

[16] Necsulea A, Soumillon M, Warnefors M, Liechti A, Daish T, Zeller U, Baker JC, Grutzner F, Kaessmann H (2014). The evolution of lncRNA repertoires and expression patterns in tetrapods. Nature.

[17] Deng L, Yang SB, Xu FF, Zhang JH (2015). Long noncoding RNA CCAT1 promotes hepatocellular carcinoma progression by functioning as let-7 sponge. J Exp Clin Cancer Res.

[18] Ding J, Lu B, Wang J, Wang J, Shi Y, Lian Y, Zhu Y, Wang J, Fan Y, Wang Z, De W Wang K (2015). Long non-coding RNA Loc554202 induces apoptosis in colorectal cancer cells via the caspase cleavage cascades. J Exp Clin Cancer Res.

[19] Fan Y, Shen B, Tan M, Mu X, Qin Y, Zhang F, Liu Y (2014). Long non-coding RNA UCA1 increases chemoresistance of bladder cancer cells by regulating Wnt signaling. FEBS J.

[20] Luo M, Li Z, Wang W, Zeng Y, Liu Z, Qiu J (2013). Long non-coding RNA H19 increases bladder cancer metastasis by associating with EZH2 and inhibiting E-cadherin expression. Cancer Lett.

[21] Yan TH, Lu SW, Huang YQ, Que GB, Chen JH, Chen YP, Zhang HB, Liang XL, Jiang JH (2014). Upregulation of the long noncoding RNA HOTAIR predicts recurrence in stage Ta/T1 bladder cancer. Tumour Biol.

[22] Zhan Y, Lin J, Liu Y, Chen M, Chen X, Zhuang C, Liu L, Xu W, Chen Z, He A, Zhang Q, Sun X, Zhao G (2016). Up-regulation of long non-coding RNA PANDAR is associated with poor prognosis and promotes tumorigenesis in bladder cancer. J Exp Clin Cancer Res.

[23] Zhuang C, Li J, Liu Y, Chen M, Yuan J, Fu X, Zhan Y, Liu L, Lin J, Zhou Q, Xu W, Zhao G, Cai Z (2015). Tetracycline-inducible shRNA targeting long non-coding RNA PVT1 inhibits cell growth and induces apoptosis in bladder cancer cells. Oncotarget.

[24] Zhan Y, Li Y, Guan B, Chen X, Chen Z, He A, He S, Gong Y, Peng D, Liu Y, Cai Z, Li X, Zhou L (2017). Increased expression of long non-coding RNA CCEPR is associated with poor prognosis and promotes tumorigenesis in urothelial bladder carcinoma. Oncotarget.

[25] Lin J, Zhan Y, Liu Y, Chen Z, Liang J, Li W, He A, Zhou L, Mei H, Wang F, Huang W (2017). Increased expression of ZEB1-AS1 correlates with higher histopathological grade and promotes tumorigenesis in bladder cancer. Oncotarget.

[26] Yang X, Song JH, Cheng Y, Wu W, Bhagat T, Yu Y, Abraham JM, Ibrahim S, Ravich W, Roland BC, Khashab M, Singh VK, Shin EJ (2014). Long non-coding RNA HNF1A-AS1 regulates proliferation and migration in oesophageal adenocarcinoma cells. Gut.

[27] Dang Y, Lan F, Ouyang X, Wang K, Lin Y, Yu Y, Wang L, Wang Y, Huang Q (2015). Expression and clinical significance of long non-coding RNA HNF1A-AS1 in human gastric cancer. World J Surg Oncol.

[28] Wu Y, Liu H, Shi X, Yao Y, Yang W, Song Y (2015). The long non-coding RNA HNF1A-AS1 regulates proliferation and metastasis in lung adenocarcinoma. Oncotarget.

[29] Liu Z, Wei X, Zhang A, Li C, Bai J, Dong J (2016). Long non-coding RNA HNF1A-AS1 functioned as an oncogene and autophagy promoter in hepatocellular carcinoma through sponging hsa-miR-30b-5p. Biochem Biophys Res Commun.

[30] Liu D, Li Y, Luo G, Xiao X, Tao D, Wu X, Wang M, Huang C, Wang L, Zeng F, Jiang G (2016). LncRNA SPRY4-IT1 sponges miR-101-3p to promote proliferation and metastasis of bladder cancer cells through up-regulating EZH2. Cancer Lett.

[31] Wilusz JE, Sunwoo H, Spector DL (2009). Long noncoding RNAs: functional surprises from the RNA world. Genes Dev.

[32] Mercer TR, Dinger ME, Mattick JS (2009). Long non-coding RNAs: insights into functions. Nat Rev Genet.

[33] Nielsen ME, Smith AB, Meyer AM, Kuo TM, Tyree S, Kim WY, Milowsky MI, Pruthi RS, Millikan RC (2014). Trends in stage-specific incidence rates for urothelial carcinoma of the bladder in the United States: 1988 to 2006. Cancer.

[34] Zhou B, Zhang P, Tang T, Liao H, Zhang K, Pu Y, Chen P, Song Y, Zhang L (2015). Polymorphisms and plasma levels of IL-27: impact on genetic susceptibility and clinical outcome of bladder cancer. BMC Cancer.

[35] Masson-Lecomte A, Rava M, Real FX, Hartmann A, Allory Y, Malats N (2014). Inflammatory biomarkers and bladder cancer prognosis: a systematic review. Eur Urol.

[36] Garcia-Closas M, Rothman N, Figueroa JD, Prokunina-Olsson L, Han SS, Baris D, Jacobs EJ, Malats N, De Vivo I, Albanes D, Purdue MP, Sharma S, Fu YP (2013). Common genetic polymorphisms modify the effect of smoking on absolute risk of bladder cancer. Cancer Res.

[37] Zhan Y, Liu Y, Wang C, Lin J, Chen M, Chen X, Zhuang C, Liu L, Xu W, Zhou Q, Sun X, Zhang Q, Zhao G (2016). Increased expression of SUMO1P3 predicts poor prognosis and promotes tumor growth and metastasis in bladder cancer. Oncotarget.

[38] Liu Y, Chen Z, He A, Zhan Y, Li J, Liu L, Wu H, Zhuang C, Lin J, Zhang Q, Huang W (2016). Targeting cellular mRNAs translation by CRISPR-Cas9. Sci Rep.

[39] Xiang JF, Yin QF, Chen T, Zhang Y, Zhang XO, Wu Z, Zhang S, Wang HB, Ge J, Lu X, Yang L, Chen LL (2014). Human colorectal cancer-specific CCAT1-L lncRNA regulates long-range chromatin interactions at the MYC locus. Cell Res.

[40] Volders PJ, Helsens K, Wang X, Menten B, Martens L, Gevaert K, Vandesompele J, Mestdagh P (2013). LNCipedia: a database for annotated human lncRNA transcript sequences and structures. Nucleic Acids Res.

[41] Chen QN, Wei CC, Wang ZX, Sun M (2017). Long non-coding RNAs in anti-cancer drug resistance. Oncotarget.

[42] Prensner JR, Sahu A, Iyer MK, Malik R, Chandler B, Asangani IA, Poliakov A, Vergara IA, Alshalalfa M, Jenkins RB, Davicioni E, Feng FY, Chinnaiyan AM (2014). The IncRNAs PCGEM1 and PRNCR1 are not implicated in castration resistant prostate cancer. Oncotarget.

[43] Ren C, Li X, Wang T, Wang G, Zhao C, Liang T, Zhu Y, Li M, Yang C, Zhao Y, Zhang GM (2015). Functions and Mechanisms of Long Noncoding RNAs in Ovarian Cancer. Int J Gynecol Cancer.

[44] Yang L, Cheng X, Ge N, Guo W, Feng F, Wan F (2017). Long non-coding RNA SPRY4-IT1 promotes gallbladder carcinoma progression. Oncotarget.

