# Supplementary material for: Long non-coding RNA HNF1A-AS1 promotes proliferation and suppresses apoptosis of bladder cancer cells through upregulating Bcl-2
Source: Oncotarget. 2017 Sep 8;8(44):76656–65. doi: 10.18632/oncotarget.20795 (PMC5652733; doi:10.18632/oncotarget.20795)
Supplement: Supplementary file 1 [file oncotarget-08-76656-s001.pdf]

Long non-coding RNA HNF1A-AS1 promotes proliferation and suppresses apoptosis of bladder cancer cells through up-regulating Bcl-2

SUPPLEMENTARY MATERIALS

Supplementary Table 1: Summary of clinicopathological features of tissues of bladder cancer

Pt No.	Sex	Age	Stage	Grade	Pt No.	Sex	Age	Stage	Grade
1	M	66	T2bN0M0	H	41	F	72	T1N0M0	L
2	M	53	T1N0M0	L	42	M	63	T3aN0M0	H
3	M	75	T2bN0M0	H	43	M	46	T1N0M0	L
4	F	64	T1N0M0	L	44	M	86	T1N0M0	L
5	M	58	T3aN0M0	H	45	M	70	T2bN0M0	H
6	M	65	T2bN0M0	H	46	M	49	T1N0M0	L
7	F	38	T3aN0M0	H	47	M	61	T3aN0M0	H
8	M	59	T2bN0M0	H	48	M	53	T2aN0M0	L
9	M	43	T3aN0M0	H	49	M	73	T2bN1M0	H
10	F	64	T2bN0M0	H	50	M	47	T2aN0M0	L
11	M	69	T1N0M0	L	51	M	77	T3aN0M0	H
12	M	72	T1N0M0	H	52	M	66	T1N0M0	L
13	F	89	T3aN0M0	L	53	F	74	T2bN0M0	H
14	M	68	T2bN0M0	H	54	F	60	T2aN0M0	H
15	F	63	T3aN0M0	H	55	M	68	T1N0M0	L
16	M	63	T2bN0M0	H	56	M	72	T2bN0M0	H
17	M	78	T2aN0M0	L	57	F	71	T1N0M0	L
18	M	70	T2aN0M0	L	58	M	54	T3aN0M0	H
19	F	41	T2aN0M0	L	59	M	60	T4aN2M0	H
20	M	59	T2bN0M0	H	60	M	52	T2aN0M0	L
21	F	73	T2aN0M0	L	61	F	67	T2aN0M0	L
22	M	67	T2bN0M0	H	62	M	71	T1N0M0	L
23	F	61	T3aN0M0	H	63	F	67	T2bN0M0	L
24	F	51	T1N0M0	L	64	M	82	T2aN0M0	L
25	M	58	T4aN3M0	H	65	M	51	T4aN0M0	H
26	M	63	T2aN0M0	L	66	M	58	T4aN0M0	H
27	M	57	T4aN0M0	H	67	M	49	T3bN1M0	H
28	M	54	T2bN0M0	H	68	M	54	T1N0M0	L
29	M	58	T4aN0M0	H	69	M	79	T1N0M0	H
30	M	63	T2aN0M0	L	70	F	73	T3aN0M0	H
31	M	50	T2bN0M0	H	71	M	69	T2bN0M0	L
32	M	73	T3bN0M0	H	72	F	77	T1N0M0	L
33	F	62	T4aN0M0	H	73	M	65	T3aN0M0	L
34	M	41	T1N0M0	L	74	M	56	T1N0M0	H

35	M	62	T4aN0M0	H	75	F	63	T2aN0M0	L
36	M	76	T2bN0M0	L	76	M	61	T4aN2M0	H
37	M	59	T4N0M0	H	77	M	49	T1N0M0	H
38	F	74	T3aN0M0	H	78	M	70	T1N0M0	L
39	F	70	T1N0M0	L	79	F	67	T2bN0M0	H
40	M	25	T1N0M0	L					

Pt No. patient number; M male; F female; Grade the World Health Organization 2004 classification; H high; L low; Stage the American Joint Committee on Cancer TNM classification.

Supplementary Table 2: The primer sequences included in this study

Name	primer sequences (5′–3′)
HNF1A-AS1:forward	TCAAGAAATGGTGGCTAT
HNF1A-AS1:reverse	GCTCTGAGACTGGCTGAA
Bcl-2:forward	GGTGGGGTCATGTGTGTGG
Bcl-2:reverse	CGGTTCAGGTACTCAGTCATCC
β-actin:forward	CATGTACGTTGCTATCCAGGC
β-actin:reverse	CTCCTTAATGTCACGCACGAT
hsa-miR-30b	GTGTAAACATCCTACACTCAGCT
U6	ACGCAAATTCGTGAAGCGTTC

Supplementary Table 3: Results of bioinformation analysis

microRNAs	lncRNAS
hsa-miR-146b-5p	HNF1A-AS1
hsa-miR-1273g-3p	HNF1A-AS1
hsa-miR-1912	HNF1A-AS1
hsa-miR-1295b-5p	HNF1A-AS1
hsa-miR-30b-5p	HNF1A-AS1
hsa-miR-6877-5p	HNF1A-AS1
hsa-miR-6747-5p	HNF1A-AS1
hsa-miR-381-5p	HNF1A-AS1
hsa-miR-3665	HNF1A-AS1
hsa-miR-514a-5p	HNF1A-AS1
hsa-miR-4512	HNF1A-AS1
hsa-miR-4689	HNF1A-AS1
hsa-miR-6858-5p	HNF1A-AS1
hsa-miR-485-5p	HNF1A-AS1
hsa-miR-624-3p	HNF1A-AS1
hsa-miR-1206	HNF1A-AS1
hsa-miR-3129-3p	HNF1A-AS1
